# UV-C irradiation as an effective tool for sterilization of porcine chimeric VP1-PCV2bCap recombinant vaccine

**DOI:** 10.1038/s41598-023-46791-9

**Published:** 2023-11-07

**Authors:** Alena Vrablikova, Martina Fojtikova, Renata Hezova, Pavlina Simeckova, Veronika Brezani, Nicol Strakova, Martin Fraiberk, Jan Kotoucek, Josef Masek, Ivan Psikal

**Affiliations:** 1 Department of Pharmacology and Toxicology, Veterinary Research Institute, Hudcova 70, 621 00 Brno, Czech Republic; 2 Faculty of Science, Charles University, Albertov 6, 128 00 Prague, Czech Republic

**Keywords:** Biotechnology, Microbiology

## Abstract

Ultraviolet irradiation is an effective method of virus and bacteria inactivation. The dose of UV-C light necessary for baculovirus inactivation by measurement of fluorescent GFP protein produced by baculovirus expression system after the irradiation of baculovirus culture in doses ranging from 3.5 to 42 J/m^2^ was determined. At a dose of 36.8 J/m^2^, only 0.5% of GFP-expressing cells were detected by flow cytometry and confocal microscopy. The stability of purified VP1-PCV2bCap protein produced by baculovirus expression system was analyzed after the irradiation at doses ranging from 3.5 to 19.3 J/m^2^. Up to the dose of 11 J/m^2^, no significant effect of UV-C light on the stability of VP1-PCV2bCap was detected. We observed a dose-dependent increase in VP1-PCV2bCap-specific immune response in BALB/c mice immunized by recombinant protein sterilized by irradiation in dose 11 J/m^2^ with no significant difference between vaccines sterilized by UV-C light and filtration. A substantial difference in the production of VP1-PCV2bCap specific IgG was observed in piglets immunized with VP1-PCV2bCap sterilized by UV-C in comparison with protein sterilized by filtration in combination with the inactivation of baculovirus by binary ethylenimine. UV-C irradiation represents an effective method for vaccine sterilization, where commonly used methods of sterilization are not possible.

## Introduction

Porcine circovirus type 2 (PCV2) is an economically important swine single stranded DNA virus pathogen spread worldwide. For the production of effective PCV2 vaccines, various techniques have been used. Many of these vaccines, especially attenuated vaccines, or recombinant protein vaccines, are genotype specific and do not have protective effect against all five major PCV2 genotypes. The production of stable and safe vaccines requires physical and/or chemical treatments to degrade potential contaminants^1^. Baculovirus-insect cell expression vector system (BEVS) is a powerful tool for production of recombinant proteins in large amounts, especially for the preparation of human and animal vaccines^2^. However, vaccines produced by BEVS may be contaminated by recombinant baculoviruses, which represent an issue for safety and registration of those recombinant vaccines. Multiple strategies of baculovirus inactivation, for example pasteurization, treatment with detergents, DNA alkylation with binary ethylenimine^3^, ionizing radiation and UV-C irradiation^1^, were previously described to minimize the risk of contaminations in biotechnological processes. UV irradiation is the effective sterilization technique against various biological contaminants, particularly viruses and bacteria^4^. Some biotechnology companies, for example Genzyme of Sanofi Pasteur, use UV irradiation as a useful strategy for the reduction of contaminants^5^.

In our previous work, we described the production and purification of chimeric VP1-PCV2bCap recombinant protein of porcine circovirus produced by BEVS as pentamers with a molecular weight of 356 kDa^6^. Due to multiple struggles with filtration and sterilization of those large aggregates, we were looking for an effective method of sterilization that could be useful also for other proteins with similar properties. We hypothesized that UV-C irradiation could be an effective tool for baculovirus inactivation where other methods for vaccine sterilization are not appropriate. Thus, we aimed to determine the dose of UV-C that could efficiently inactivate baculovirus without damaging the VP1-PCV2bCap recombinant protein.

In this work, we describe the purification of chimeric VP1-PCV2bCap recombinant protein complexes by affinity chromatography and analysis of the stability and immunogenicity of this protein after the irradiation by UV-C light and effect of UV-C light on the degradation of baculovirus as a possible contaminant of recombinant vaccines produced by BEVS. VP1-PCV2bCap represents a modern and safe immunizing agent capable of inducing a strong humoral immune response against PCV2 infection with significant neutralizing activity. Moreover, this chimeric antigen containing VP1-PCV2bCap protein sequences based on the mouse polyomavirus (MPyV) has a so-called DIVA (differentiating infected from vaccinated animals) vaccine potential, which can induce an immune response against the mouse polyoma VP1 protein and distinguish between PCV2 naturally infected and vaccinated animals.

## Results

**Figure 1:**
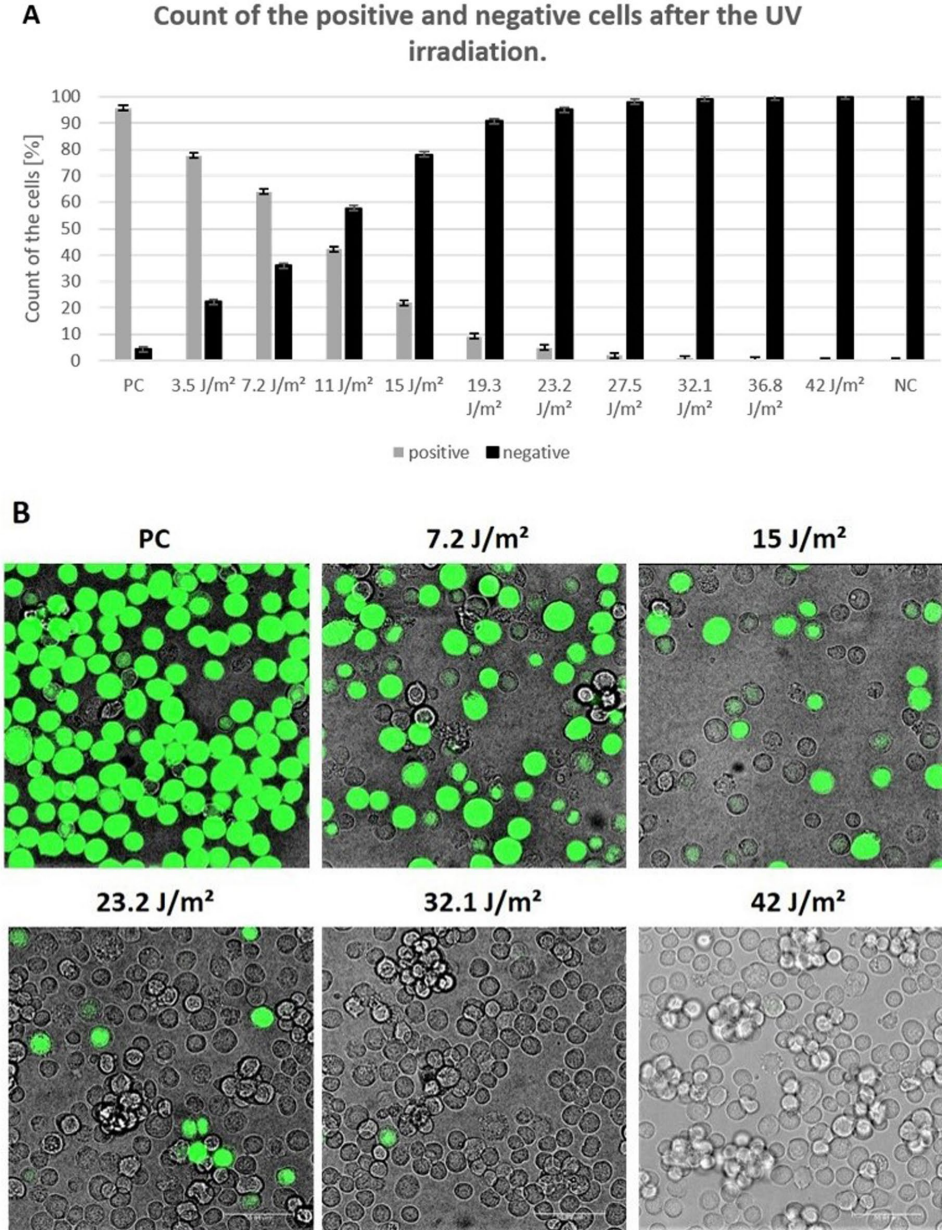
Effect of UV-C light on the viability of Bac-GFP after infection of SF9 cells monitored by flow cytometry (**A**) and confocal microscopy (**B**). SF9 cells infected with Bac-GFP irradiated by UV-C in doses 3.5 J/m^2^, 7.2 J/m^2^, 11 J/m^2^, 15 J/m^2^, 19.3 J/m^2^, 23.2 J/m^2^, 27.5 J/m^2^, 32.1 J/m^2^, 36.8 J/m^2^ and 42 J/m^2^ were incubated for 36 h. Fluorescence of expressed GFP protein was detected. *Positive control (PC)*—SF9 cells infected by Bac-GFP without irradiation. *Negative control (NC)*—uninfected SF9 cells.

**Figure 2:**
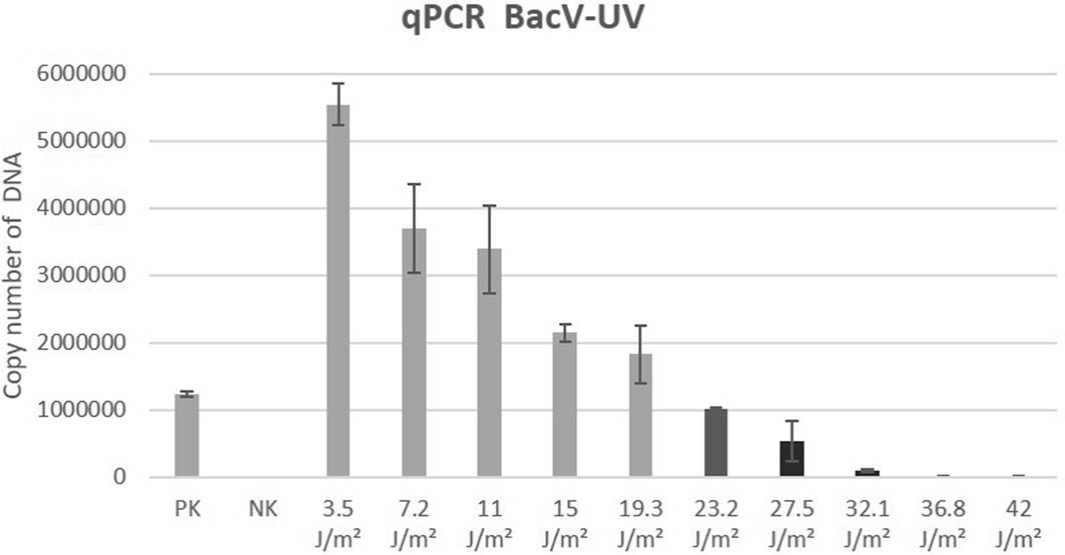
The effect of UV-C light on baculovirus DNA damage. DNA was isolated from SF9 cells infected with Bac-GFP irradiated by UV-C in doses 3.5 J/m^2^, 7.2 J/m^2^, 11 J/m^2^, 15 J/m^2^, 19.3 J/m^2^, 23.2 J/m^2^, 27.5 J/m^2^, 32.1 J/m^2^, 36.8 J/m^2^ and 42 J/m^2^ after the incubation for 36 h. The copy number of DNA was analyzed by real-time PCR. *Positive control (PC)*—SF9 cells infected by Bac-GFP without irradiation. *Negative control (NC)*—uninfected SF9 cells.

SF9 cells were infected with recombinant baculovirus containing Green Fluorescent Protein gene (Bac-GFP) irradiated by UV-C light at doses ranging from 3.5 to 42 J/m^2^. The effect of UV light on the viability of Bac-GFP was evaluated by monitoring GFP-fluorescent SF9 cells by flow cytometry (Fig. 1A) and confocal microscopy (Fig. 1B), where the fluorescent SF9 cells corresponded to cells infected by surviving Bac-GFP capable to express GFP protein. We detected Bac-infected cells expressing GFP protein in cultures treated with Bac-GFP irradiated by UV-C at doses ranging from 3.5 to 32.1 J/m^2^. The percentage of Bac-GFP-positive cells gradually decreased with increasing levels of UV-C irradiation. The result was comparable for both methods. In addition to decreasing counts of fluorescent SF9 cells with increasing dose of UV-C irradiation, we also observed gradual reduction of fluorescence intensity that probably corresponded to structural changes of GFP protein after higher doses of irradiation. Bac-GFP irradiation at a dose of 36.8 J/m^2^ UV-C light resulted in the detection of less than 0.5% of Bac-GFP-infected cells and no infected cells were observed after Bac-GFP irradiation at 42 J/m^2^. The effect of UV-C light on baculovirus DNA damage at the molecular level was verified by real-time PCR in samples of DNA isolated from SF9 cells infected with Bac-GFP and irradiated by UV-C light at doses ranging from 3.5 to 42 J/m^2^ (Fig. 2). The amount of DNA gradually decreased with the dose of UV-C from 3.5 to 27.5 J/m^2^ and dramatically reduced at the dose of 32.1 J/m^2^ and higher.

**Figure 3:**
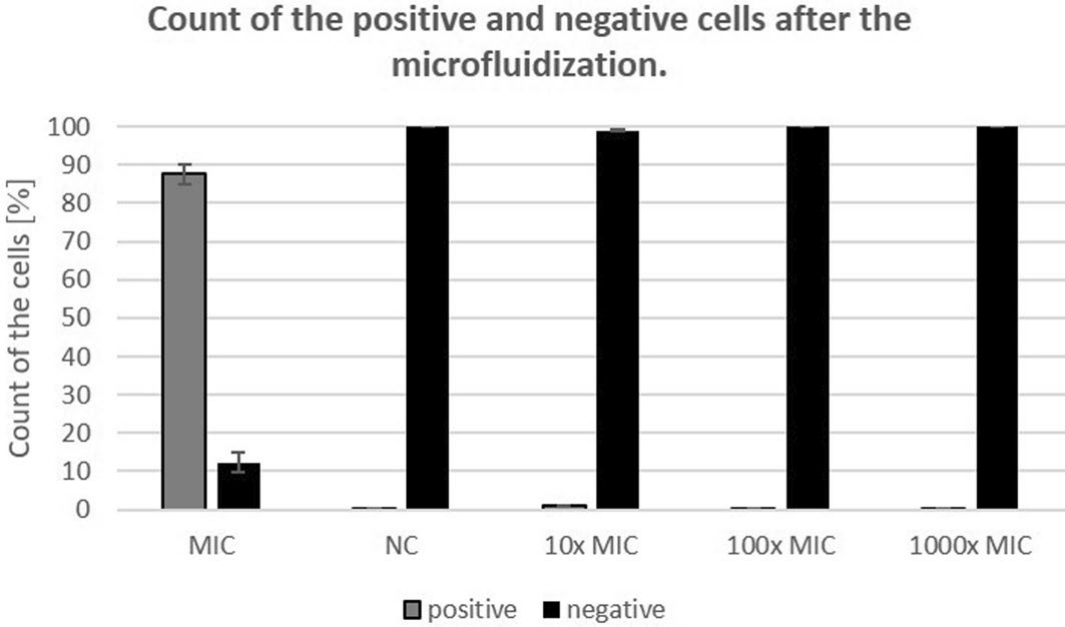
Virulence of Bac-GFP titer after microfluidization monitored by flow cytometry. *MIC*—SF9 cells infected by undiluted Bac-GFP after the microfluidization; *NC*—negative control (uninfected SF9 cells); 10 × MIC—SF9 cells infected by 10 × diluted Bac-GFP after the microfluidization; 100 × MIC—SF9 cells infected by 100 × diluted Bac-GFP after the microfluidization; 1.000 × MIC—SF9 cells infected by 1.000 × diluted Bac-GFP after the microfluidization.

To determine the virulence of baculovirus after microfluidization, which is commonly used method for cell lysis and further isolation of protein of interest, we applied the lysates from Bac-GFP infected SF9 cells to non-infected SF9 cells. Infectivity of Bac-GFP after the lysis by microfluidization was analyzed by flow cytometry. Less than 1% of positive cells were detected in samples of cells infected by Bac-GFP diluted 10 ×, 100 × or 1.000 × (Fig. 3). Less than 0.5% of positive cells were detected after the process of microfluidization complemented with DENARASE treatment and purification across the CoNTA column (data not shown).

**Figure 4:**
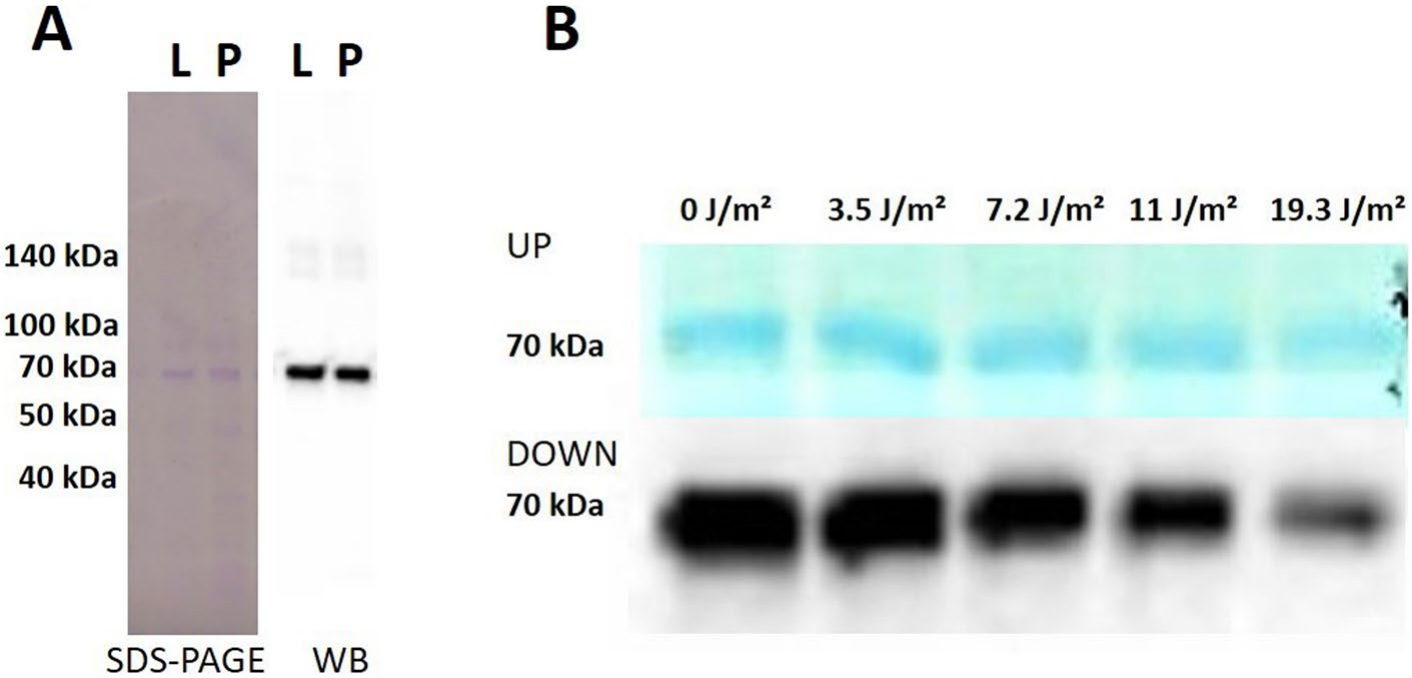
Analysis of the VP1-PCV2bCap stability after the UV-C irradiation. (**A**) Purified protein was separated on SDS-PAGE followed by Coomassie Brilliant Blue staining (SDS-PAGE) or analyzed by Western blot (WB) detected by Anti-6X His tag HRP antibody. *L* cell lysate, *P* purified protein. (**B**) Purified VP1-PCV2bCap protein irradiated by UV-C light at a dose of 3.5 J/m^2^, 7.2 J/m^2^, 11 J/m^2^, and 19.3 J/m^2^, separated on SDS‐PAGE (UP) and analyzed by western blot developed with Anti-6X His tag HRP antibody (DOWN). The amount of irradiated protein was partially decreased after 11 J/m^2^ and each subsequent UV-C irradiation. 0 J/m^2^—VP1-PCV2b before irradiation.

VP1-PCV2bCap protein was expressed in SF9 cells and purified by CoNTA affinity chromatography in the high purity (Fig. 4A). We were able to obtain 2 mg of purified protein from 300 mL of cell culture. The stability of purified protein after the irradiation by UV-C light in dose 3.5 J/m^2^, 7.2 J/m^2^, 11 J/m^2^, and 19.3 J/m^2^ was analyzed by SDS-PAGE and western blot with antibody detecting His-tag of purified protein. The protein stayed relatively stable after the irradiation at a dose up to 11 J/m^2^ of UV-C. Further increase of UV-C dose led to partial breakdown of the protein (Fig. 4B). Therefore, the dose of 11 J/m^2^ of UV-C that can substantially decrease the viability of baculovirus and does not affect VP1-PCV2bCap protein stability was applied to sterilize VP1-PCV2bCap protein mixture which was further tested on animals.

**Figure 5:**
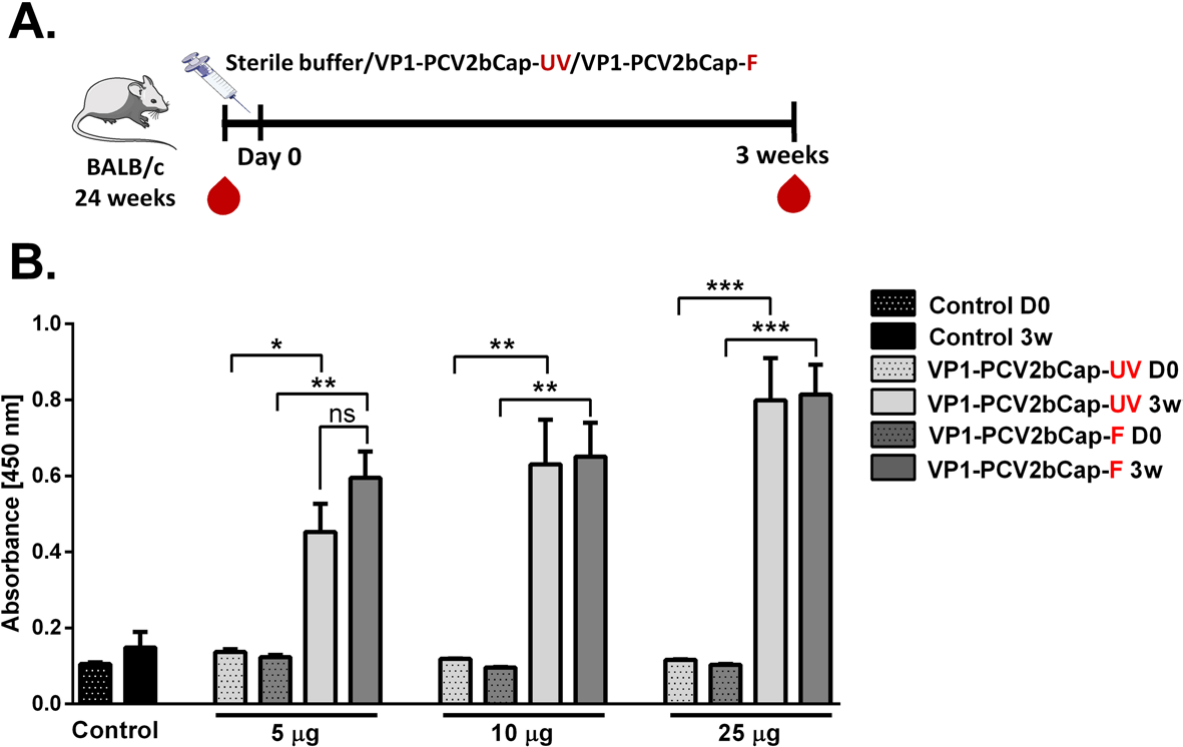
Comparison of VP1-PCV2bCap-specific antibody response in mice induced by vaccine containing purified protein sterilized by UV-C irradiation or filtration. Six groups of mice were immunized subcutaneously with mixture of purified VP1-PCV2bCap protein in combination with Emulsigen (10%) sterilized by UV-C irradiation in dose 5 µg, 10 µg or 25 µg or by filtration in the same doses. Control mice were injected with a sterile buffer. Sera were collected directly before immunization and 3 weeks after the immunization. (**A**) Immunization scheme. (**B**) Levels of VP1-PCV2bCap-specific IgG antibodies at different time points (D0—day 0/before immunization, 3w—3 weeks) and doses of antigen. *P < 0.05, **P < 0.01, ***P < 0.001 compared to the respective control before immunization (D0) vs. after immunization at 3 weeks. Mean ± SEM. One-way ANOVA with Bonferroni post hoc test.

The immunogenicity of the protein mixture sterilized by UV-C irradiation in dose of 11 J/m^2^ was analyzed by ELISA 3 weeks after the immunization of mice by antigen containing 5 µg, 10 µg, or 25 µg of purified VP1-PCV2bCap protein supplemented with Emulsigen (10%) as an adjuvant. Immunogenicity of VP1-PCV2bCap sterilized by irradiation was compared to VP1-PCV2bCap sterilized by filtration and administered at the same doses (Fig. 5A). High amounts of specific antibodies were detected in all immunized mice 3 weeks after immunization. The levels of specific IgG antibodies were increased in a protein dose-dependent manner with the most robust immune response in mice vaccinated with 25 µg of purified VP1-PCV2bCap protein. No significant differences were observed between groups of mice immunized with protein mixture sterilized by UV-C irradiation compared to mice immunized with protein sterilized only by filtration (Fig. 5B).

**Figure 6:**
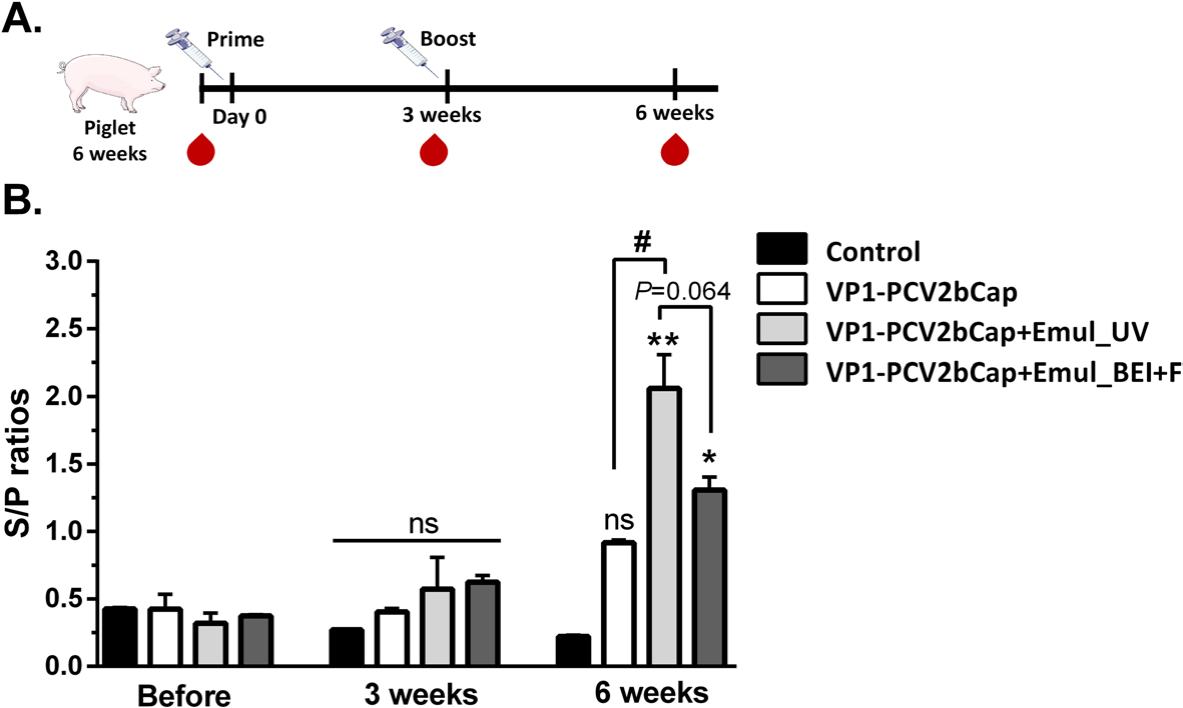
Comparison of VP1-PCV2bCap-specific antibody response in piglets induced by mixture of purified protein sterilized by UV-C irradiation or filtration after the BEI inactivation. Piglets (n = 4) were immunized intramuscularly twice (prime at day 0 and booster at 3 weeks) with 50 µg of purified VP1-PCV2bCap protein alone (without sterilization) or VP1-PCV2bCap in combination with Emulsigen (10%; Emul) sterilized by UV-C irradiation or filtration after the inactivation by BEI. Control piglets (n = 2) were injected only with a sterile buffer. Sera were collected before vaccination, at 3 weeks (before booster) and 6 weeks after the first vaccination. (**A**) Immunization scheme. (**B**) Levels of VP1-PCV2bCap-specific IgG antibodies at different time points. *P < 0.05, **P < 0.01 compared to the non-immunized control at 6 weeks. ^#^*P* < 0.05 indicates significant difference between VP1-PCV2bCap protein (without inactivation) vs. VP1-PCV2bCap + Emulsigen 10% with UV-C irradiation at 6 weeks. Mean ± SEM. One-way ANOVA with Bonferroni post hoc test.

The efficacy of VP1-PCV2bCap protein sterilized either by UV-C (11 J/m^2^) or by binary ethylenimine (BEI) in combination with filtration was then verified in the pigs by the prime/boost immunization of 6-week-old piglets. Humoral immune response was monitored for 6 weeks (Fig. 6A). While the immune response after the first dose was poor with no difference between piglets immunized with VP1-PCV2bCap irradiated and VP1-PCV2bCap filtered, the levels of specific antibodies increased markedly 3 weeks after the booster. Interestingly, VP1-PCV2bCap protein mixture sterilized by UV-C irradiation elicited significantly higher levels of specific IgG antibodies than protein mixture inactivated by BEI and sterilized by filtration (Fig. 6B).

**Figure 7:**
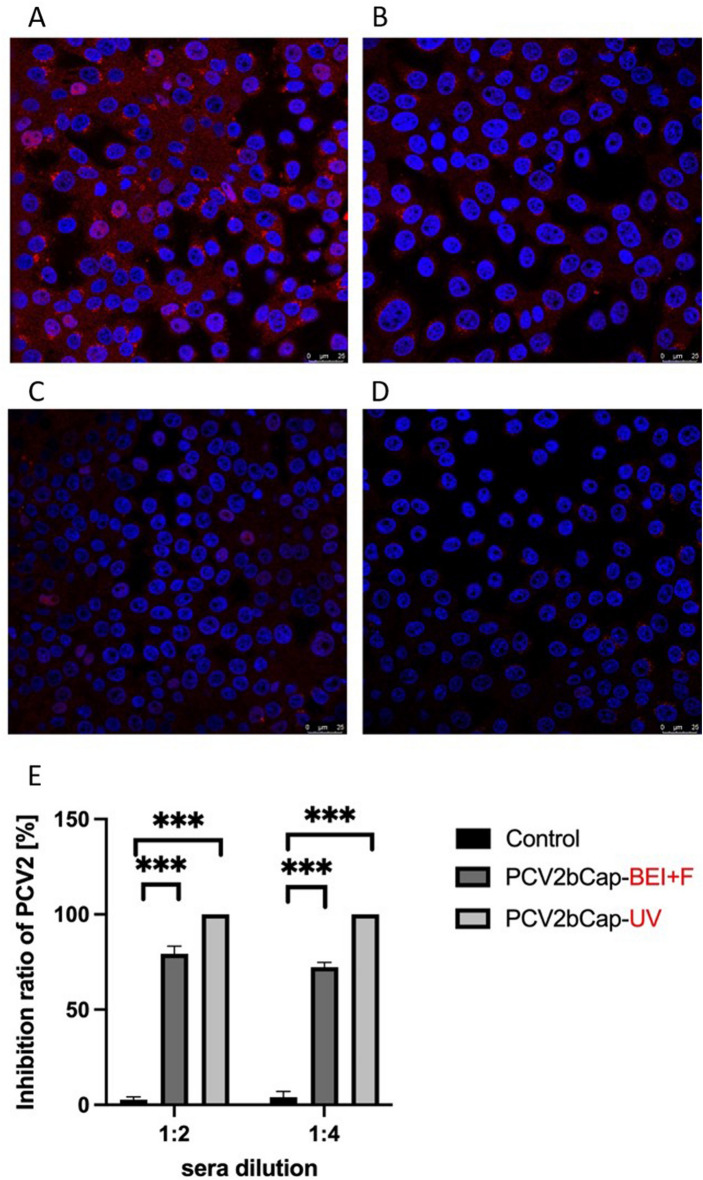
The neutralization effect of VP1-PCV2bCap antibodies from piglets immunized with VP1-PCV2bCap protein mixture on PCV2 infected PK-15 cells. The cells were stained with anti-PCV2Cap H9 Alexa Fluor-647 (red) and the nuclei were stained with Hoechst (blue). The representative overlay images (pink) from 3 independent repetitions in 6 areas per well. (**A**) Positive control cells were infected with PCV2 Stoon 1010 virus and treated with sera of control group. (**B**) The cells infected with PCV2 Stoon 1010 virus treated with sera of piglets immunized with purified VP1-PCV2bCap protein mixture sterilized by UV-C irradiation. (**C**) The cells infected with PCV2 Stoon 1010 virus treated with sera of piglets immunized with purified VP1-PCV2bCap protein mixture sterilized by filtration after the BEI inactivation. (**D**) Non-infected cells were used as a negative control. (**E**) PCV2 positive cells were counted and the inhibition ratio of PCV2 infection was compared. ***P < 0.001 differences between positive control cells infected with PCV2 Stoon 1010 virus treated with sera of control group and cells infected with PCV2 Stoon 1010 virus treated with sera of piglets immunized withVP1-PCV2bCap protein mixture supplemented with Emulsigen (10%) UV-C sterilized or sterilized by filtration after the BEI inactivation. ANOVA with Bonferroni post hoc test.

Production of effective neutralizing antibodies was determined by neutralization assay based on the infection of porcine PK-15 kidney cells by mixture of PCV2 Stoon 1010 virus inoculum with inactivated piglet sera collected at 6 weeks after the first immunization. As positive control PK-15 cells treated with serum of control group of piglets was used. Sera of piglets immunized by mixture of purified protein sterilized by UV-C irradiation showed a better neutralization effect as sera of piglet immunized by protein mixture sterilized by filtration after the inactivation by BEI (Fig. 7).

## Discussion

UV-C irradiation is simple, effective, and safe sterilization method to dispose of bacteria and viruses^7^. This method was tested for the inactivation of many human and animal viruses. The minimum dose of irradiation used for inactivation of microorganisms depends on the morphological features of microorganisms and probably on the distance from the source of irradiation. As a rule, nonenveloped viruses are more resistant to ultraviolet irradiation than enveloped viruses^8,9^. However, this is not a strict guideline, for example, porcine parvovirus (PPV) a nonenveloped virus is highly sensitive to UV-C requiring only 2.4 J/m^2^ dose of irradiation for complete inactivation^10^, whereas porcine circovirus (PCV) another nonenveloped virus requires about 24 J/m^2^^11^. Baculoviruses represent double stranded DNA viruses highly resistant to UV-C irradiation due to their ability to repair genomes during replication processes within the host cells^12^. In the case of Bac-GFP, a dose of 36.8 J/m^2^ completely inactivated baculoviruses, and no expression of GFP protein was detected by flow cytometry and confocal microscopy. Similar results were observed after the real-time PCR analysis. We detected a gradually decreased amount of baculovirus DNA up to dose 27.5 J/m^2^ of irradiation and dramatic decreased concentration of DNA at the dose of 32.1 J/m^2^ and higher. This is in agreement with previously published data, which describes false-positive signals by real-time PCR when the damage of the viral genome is less than 1:1^13^. Real-time PCR is also not capable to distinguish between infectious and noninfectious viral genomes^14^. Importantly, we showed that the virulence of baculovirus is radically decreased after microfluidization step during the protein isolation process. However, sterilization of the final product by an appropriate method is necessary to inactivate the residual baculovirus which could potentially be present in the protein mixture.

Irradiation by UV-C light leads to the degradation of pathogens and nucleic acids with less damage of surface antigenic epitopes of produced protein and minimal changes in immune response to the vaccine in comparison to gamma radiation or chemical sterilization^1^. Germicidal UV light (200–300 nm) produces photodimeric lesions between adjacent pyrimidine nucleotides in deoxy and ribonucleic acids of bacteria, viruses, and protozoa. These changes block transcription and replication processes causing the inactivation of the microorganisms^13^.

Many structural and/or functional modifications of proteins caused by UV-C irradiation lead to the breaking of disulfide bonds, cross-linking, aggregation, fragmentation, oxidation, or deamination of proteins, and often a subsequent reduction in the activity of proteins^5^. In the case of our chimeric protein VP1-PCV2bCap, increasing the dose of UV-C light led to partial degradation of the protein detected by lowered band density in western blot analysis. No fragments of chimeric protein evaluated by anti-His tag antibody were detected. However, minimal damage to chimeric protein was observed after irradiation with the dose of 11 J/m^2^ UV-C light. In contrast to the irradiation method, sterilization by filtration led in our case to the loss of considerable amount of purified chimeric protein due to its high molecular weight (365 kDa) and retention of the protein by filter (Supplementary information, Fig. S3). In addition, our data suggest that a substantial part of baculoviruses are degraded after the lysis of the cells by microfluidization. No live baculovirus was observed in elution after the purification through the CoNTA column and so a minimal dose of UV-C irradiation is necessary for the total sterilization of the final product. To compare the immunological effectiveness of VP1-PCV2bCapprotein mixture sterilized by UV-C irradiation vs. filtration, BALB/c mice were immunized in the one-step vaccination protocol. The immune response was dose-dependent, but no significant differences were observed between the two types of diversely sterilized protein mixtures. In the case of in vivo study on piglets, we used UV-C irradiation or filtration in combination with BEI for the complete inactivation of residual baculoviruses in a protein mixture. We observed a significant increase of specific antibody titers in the group of piglets immunized by protein mixture sterilized by UV-C irradiation 3 weeks after the booster, but a markedly lower response in the group vaccinated with VP1-PCV2bCap sterilized by filtration in combination with BEI inactivation. BEI has been widely tested for the inactivation of viral vaccines^15–17^. Valero et al. described the lower neutralization effect of antibodies produced by vaccine inactivated by BEI^15^. This is in agreement with our in vivo study on piglets and the results from the neutralization assay, where we observed a better neutralization effect of antibodies in sera of piglets immunized by protein mixture sterilized by UV-C irradiation in comparison to those vaccinated by protein mixture sterilized by filtration after the inactivation by BEI. We assume that a greater neutralization effect may be due to an overall higher level of IgG specific antibodies in the sera of piglets immunized with VP1-PCV2bCap protein mixture sterilized by UV-C irradiation. The neutralization effect can also correlate with other immune responses. Comparison of T cell and B cell-mediated immune responses to immunization and their contribution to protection would provide more information about the effectiveness of vaccines. Still, UV-C light irradiation represents a promising sterilization strategy for the preparation of recombinant protein vaccines applicable in veterinary practice.

## Conclusion

Sterilization of recombinant protein vaccine by UV-C irradiation is a promising strategy for the preparation of large chimeric protein antiviral vaccines, where filtration, pasteurization, alkylation, and treatment with detergents are not possible due to specific properties of chimeric proteins. UV-C irradiation is an effective method of baculovirus inactivation and an appropriate method of sterilization as a key step for the production of safe chimeric recombinant vaccines.

## Materials and methods

### Preparation of recombinant plasmid with GFP (Bac-GFP)

Construction of recombinant plasmid was described previously^18^. Briefly, C-terminal fragment of VP3 protein was amplified by PCR (forward primer: 5′-CATCAGCGAGCTCAGGGTACTC-3′ and reverse primer: 5′-TTAGAGGATCCTTAGAGACGCCGCTT-3′). The PCR fragment and pEGFP-C2 plasmid (Clontech, Palo Alto, CA) were cut with restriction enzymes Sac I and BamH I and ligated together to generate pEGFP-t-VP3 plasmid. EGFP-t-VP3 sequence was amplified by PCR (forward primer: 5′-AGATAGGATCCACCATGGTGAGCAAG-3′ and reverse primer: 5′-TTAGAGGATCCTTAGAGACGCCGCTT-3′). The PCR product was cut with restriction enzymes Bgl II and BamH I and inserted into the pAcDB3/VP1 plasmid (gift from Drs. T. Ramqvist and T. Dalianis, Karokinska Institut, Department of Oncology and Pathology, Sweden). Recombinant baculovirus Bac-GFP was prepared and purified by plaque assays as was described previously^19^.

### Analysis of Bac-GFP inactivation by flow cytometry and confocal microscopy

Bac-GFP (10^7.8^ IFU/ml; 60 ml) in SFM900II medium (Gibco, USA) was irradiated by Steripen UltraLight (Hydro-Photon, Inc., USA) in dose 3.5 J/m^2^, 7.2 J/m^2^, 11 J/m^2^, 15 J/m^2^, 19.3 J/m^2^, 23.2 J/m^2^, 27.5 J/m^2^, 32.1 J/m^2^, 36.8 J/m^2^ and 42 J/m^2^ of UV-C light. 1.5 million SF9 cells (Gibco) were infected with 100 µl of irradiated Bac-GFP and incubated at 27.5 °C in the shaking incubator at 200 rpm. After 36 h, the expression of the GFP protein was analyzed by Amnis CellStream Flow Cytometer (Luminex, USA) and confocal microscope (Leica Microsystems, Wetzlar, Germany). For flow cytometry 1 mL of the cell suspension was centrifuged for 1 min at 500 **µ**g, resuspended in 1 mL sterile PBS and centrifuged again. Pellets were resuspended in 0.5 mL of sterile PBS and analyzed immediately. For confocal microscopy, 100 µL of cell suspension was pipetted into sterile µ-Slide 8-Well chambers (Ibidi GmbH, Germany) and analyzed immediately.

### Analysis of Bac-GFP inactivation by real-time PCR

Bac-GFP (10^7.8^ IFU/ml; 60 mL) in SFM900II medium (Gibco) was irradiated by Steripen UltraLight (Hydro-Photon, Inc.) in dose 3.5 J/m^2^, 7.2 J/m^2^, 11 J/m^2^, 15 J/m^2^, 19.3 J/m^2^, 23.2 J/m^2^, 27.5 J/m^2^, 32.1 J/m^2^, 36.8 J/m^2^ and 42 J/m^2^ of UV-C light. 1.5 million SF9 cells (Gibco) were infected with 100 µl of irradiated Bac-GFP and incubated at 27.5 °C in the shaking incubator at 200 rpm. After 36 h, samples were two times freezed at – 80 °C for 15 min and incubated at 80 °C for 5 min. Then, 10 sample volumes of buffer PBS supplemented with 1% Triton X100 were added to the samples. The samples were again two times freezed at – 80 °C for 15 min and incubated at 80 °C for 5 min. The samples in the amount of 0.3 µl were used for the RT-PCR reaction with 2 × qPCR Master Mix (Ambion GmbH, Germany), primers (forward primer: 5′-CGGCGTGAGTATGATTCTCAAA-3′ and reverse primer: 5′-ATGAGCAGACACGCAGCTTTT-3′) and probe (5′-AAAAGTCTACGTTCACCACGCGCCAAA-3′) and analyzed by QuantStudio Real-Time PCR System (Applied Biosystems, USA).

### Analysis of Bac-GFP culture titer and virulence after the microfluidization

Bac-GFP was 10 ×, 100 × and 1.000 × diluted with SFM900II medium. 100 µL of Bac-GFP culture was added into 15 million SF9 cells in SFM900II and cultivated for 36 h by shaking at 27.5 °C at 200 rpm. SF9 cells were then harvested, and pellet was frozen at − 80 °C overnight. Pellet was resuspended in 30 ml native lysis buffer (50 mmol L^−1^ Tris‐HCl, 150 mmol L^−1^ NaCl, pH 7.4) supplemented with complete, EDTA-free Protease Inhibitor Cocktail (Merck, USA) and lysed by Microfluidizer Processor Cell Disruptor Homogenizer (New Life Scientific, USA) cooled on ice. 100 µL of the lysate after the microfluidization was added into 1.5 million SF9 cells in SFM900II and cultivated for 2 h by shaking at 27.5 °C at 200 rpm. Cultures were centrifuged for 10 min at 500 µg, resuspended in fresh SFM900II and repeatedly centrifuged (10 min, 500 µg). Pellets were resuspended in fresh SFM900II and incubated for 36 h by shaking at 27.5 °C at 200 rpm. For flow cytometry, 1 mL of the cell suspension was centrifuged for 1 min at 500 µg, resuspended in 1 mL sterile PBS and centrifuged again. Pellets were resuspended in 0.5 mL sterile PBS and analyzed immediately. For confocal microscopy, 100 µL of cell suspension was pipetted into sterile µ-Slide 8-Well chambers (Ibidi GmbH) and analyzed immediately.

### Purification of chimeric protein from SF9 insect cells

Insertion of sequence of porcine circovirus 2b (PCV2b) capsid protein into pFastBacI – VP1_DAɅ7_ transfer vector, preparation of recombinant baculovirus, generation of high-titer viral stocks and protein production were described previously^6,20^.

SF9 cell pellet containing VP1-PCV2bCap recombinant protein was resuspended in native lysis buffer (20 mmol L^−1^ Tris‐HCl, 150 mmol L^−1^ NaCl, pH 7.6) supplemented with cOmplete, EDTA-free Protease Inhibitor Cocktail (Merck) and lysed by Microfluidizer Processor Cell Disruptor Homogenizer (New Life Scientific) cooled on ice. Cell lysate was centrifuged at 10.000 µg for 30 min and supernatant was treated with 180 U DENARASE (c-LEcta GmbH) and 2 µl of 1 M MgCl_2_ per ml of supernatant and incubated for 1 h at 37 °C. Supernatant was centrifuged at 10.500 µg for 10 min and chimeric VP1-PCV2bCap protein was purified under native conditions using CoNTA agarose (Merck) according to the manufacturer’s instructions. The purified VP1-PCV2bCap was further used for evaluation of irradiation dose–response effect, protein analysis, and immunization of the animals. The irradiation was performed as a final step after protein purification to inactivate residual baculovirus.

### Analysis of the VP1-PCV2bCap stability after the irradiation by Steripen UltraLight

Purified VP1-PCV2bCap protein (60 mL) in concentration 0.2 mg/mL was irradiated by Steripen UltraLight in dose 3.5 J/m^2^, 7.2 J/m^2^, 11 J/m^2^, and 19.3 J/m^2^ of UV-C light according to the manufacturer’s instructions. VP1-PCV2bCap proteins were analyzed by SDS-PAGE and Western blot.

#### SDS-PAGE and Western blot analysis

The cell lysate, purified protein with or without irradiation were separated on 10% SDS-PAGE stained with Coomassie Brilliant Blue R‐250 or blotted onto polyvinylidene difluoride (PVDF) membrane (Millipore, USA). The PVDF membrane was blocked overnight with 3% non‐fat milk and developed with Anti-6X His tag HRP antibody (ABCAM, UK; dilution 1:1.000) followed by the detection using GE Healthcare Amersham ECL Prime Western Blotting Detection Reagent (Thermo Fisher, USA) and analyzed with Azure Biosystems C300 (Azure Biosystems, Inc., USA) and cSeries Capture Software.

### Immunization of animals

Experiments were conducted according to the principles enunciated in the Guide for the Care and Use of Laboratory Animals issued by the Czech Society for Laboratory Animal Science and reviewed by the Ethical committee and approved by the Ministry of Agriculture of the Czech Republic (permit no. MZE-2334 and 9487/2019-3). Water and standard pellet diet were given ad libitum.

Mice experiments were performed on 24-week-old female BALB/c mice (purchased from Laboratory Animal Breeding and Experimental Facility, MUNI, Czech Republic). Mice were divided into seven groups (3 mice per group) and immunized subcutaneously (s.c.):

1. Group immunized by 5 μg purified VP1-PCV2bCapprotein in sterile buffer (50 mmol L^−1^ Tris‐HCl, 300 mmol L^−1^ NaCl, pH 7.4) supplemented with Emulsigen (10%) as adjuvant, sterilized by UV-C irradiation in a dose of 11 J/m^2^.
2. Group immunized by 10 μg purified VP1-PCV2bCapprotein in sterile buffer supplemented with Emulsigen (10%) as adjuvant, sterilized by UV-C irradiation in a dose of 11 J/m^2^.
3. Group immunized by 25 μg purified VP1-PCV2bCapprotein in sterile buffer supplemented with Emulsigen (10%) as adjuvant, sterilized by UV-C irradiation in a dose of 11 J/m^2^.
4. Group immunized by 5 μg purified VP1-PCV2bCapprotein in sterile buffer supplemented with Emulsigen (10%) as adjuvant, sterilized by filtration (pore size 0.45 µm).
5. Group immunized by 10 μg purified VP1-PCV2bCapprotein in sterile buffer supplemented with Emulsigen (10%) as adjuvant, sterilized by filtration.
6. Group immunized by 25 μg purified VP1-PCV2bCapprotein in sterile buffer supplemented with Emulsigen (10%) as adjuvant, sterilized by filtration.
7. Control group injected only with a sterile buffer.

Blood of mice was collected before immunization and 3-weeks after the immunization.

Piglet experiments were performed on 6-week-old piglets (purchased from Bioprodukt Knapovec a.s., Czech Republic). Piglets were divided into three groups:

1. 4 piglets immunized with 50 μg of purified VP1-PCV2bCap protein in a sterile Dulbecco’s Phosphate Buffered Saline (Sigma-Aldrich) supplemented with Emulsigen (10%) as adjuvant sterilized by UV-C irradiation in dose 11 J/m^2^.
2. 4 piglets immunized with 50 μg of purified VP1-PCV2bCap protein in sterile Dulbecco’s Phosphate Buffered Saline (Sigma Aldrich) supplemented with Emulsigen (10%) as adjuvant. Co-produced baculoviruses were inactivated by binary ethylenimine (BEI) in concentration 10 mM for total contact time of 24 h at 37 °C. The BEI activity was neutralized with 15 mM of sodium thiosulfate. Final mixture was sterilized by filtration (pore size 0.45 µm).
3. 2 piglets immunized with VP1-PCV2bCap protein in a sterile Dulbecco’s Phosphate Buffered Saline without adjuvant and baculovirus inactivation, filtered through 0.45 µm filter.
4. 2 control piglets injected only with a sterile Dulbecco’s Phosphate Buffered Saline

Piglets were immunized twice intramuscularly (i.m.) with a booster applied at 3-weeks after the first immunization. Blood was collected before immunization, 3-weeks, and 6-weeks after the first immunization.

### ELISA analyses

Indirect ELISA analyses were realized using commercial kit INgezim Circo IgG according to the manufacturer’s instructions (Eurofins Technologies Ingensa, Spain). Briefly, 100 µL of sera were diluted at a 1:200 ratio, transferred to the ELISA plate and incubated for 1 h at room temperature (RT). ELISA plate was washed 4 times and 100 µL of conjugate were pipetted to each well of the plate and incubated for 30 min at RT [in the case of mice Goat-anti mouse IgG (Whole Molecule) Peroxidase Conjugate (Sigma Aldrich) in a dilution of 1:6.000 was pipetted to each well of the plate and incubated at RT for 2 h]. After 6-time wash, 100 µL of substrate were pipetted to the each well and incubated at RT until reaction was stopped by 100 µL stop solution. Optical density was analyzed at 450 nm by Gen5 Data Analysis Software (BioTek, USA).

### Preparation of conjugate antibody

The monoclonal antibody PCV2Cap H9 (Exbio, Czech Republic) was labelled with the fluorescent probe Alexa Fluor 647 NHS Ester (Thermo Fisher). The fluorescence dye was dissolved in anhydrous dimethyl sulfoxide (DMSO) to the final concentration of 10 µg/µL. 500 µL of the monoclonal antibody PCV2Cap H9 at a concentration of 2 mg/mL in PBS was mixed with 50 µL of the 1 M solution of sodium bicarbonate (pH 8.5) and 2 µL of the fluorescence dye solution was added to the final mixture. The reaction was carried out at RT and constant stirring for 1 h and the unbound dye was removed using a spin column at 1.000×*g* for 2 min.

### Virus neutralization assay

PCV-free PK-15 cells (kindly provided by Prof. Gordon Allan, The Queen’s University Belfast, Belfast, Northern Ireland, UK) were maintained in Minimum Essential Medium (MEM, Biowest, France) supplemented with 10% fetal bovine serum (FBS), 1% penicillin, and 1% streptomycin (complete medium; all from Sigma Aldrich) at 37 °C in a humidified atmosphere containing 5% CO_2_. Reference strain PCV2 Stoon 1010 (kindly provided by Prof. Gordon Allan, The Queen’s University Belfast, Belfast, Northern Ireland, UK) was passaged several times in PK-15 cells maintained in 2% FBS, 1% penicillin, and 1% streptomycin culture medium for 72 h at 37 °C.

PK-15 cells were seeded into the µ-Slide 8-Well chambers (Ibidi GmbH) in volume 300 μL/well at a density of 2.5 × 10^5^ cells/mL in a complete MEM medium. After 24 h, cell culture medium was replaced with 0.03% nonionic surfactant Tween 20 (Sigma Aldrich) in a serum-free medium for 23 h. To allow attachment of antibodies to virus, diluted sera (1:2 and 1:4) of piglets immunized by 50 μg purified VP1-PCV2bCapprotein with Emulsigen (10%) [sterilized by UV-C irradiation (group 1) or sterilized by filtration in combination with BEI (group 2) or controls (group 3)] were mixed in equal volume of 100 µL with viral inoculum (10^–3.2^ TCID_50_ mL^–1^) PCV2 Stoon 1010 and incubated at RT for 1 h. The mixture was then added to the pretreated cells with nonionic surfactant Tween 20 and incubated for 1 h at 37 °C_._ PK-15 cells were washed and incubated for 72 h in cell culture medium with 2% FBS^21^. The untreated and treated cells were fixed in 4% v/v paraformaldehyde (Sigma Aldrich) at RT for 15 min and permeabilized by 0.05% Triton X-100 (Serva, Germany) in PBS at RT for 5 min. The cells were washed three times with PBST_20_ and blocked with 4% bovine serum albumin in PBS for 30 min at 37 °C. 125 × diluted Alexa Fluor 647 conjugate primary antibodies (PCV2Cap H9, Exbio, Czech Republic) in blocking buffer (100 µL/well) were added to PK-15 cells and incubated for 1 h at 37 °C. Cells were washed 3 times with PBST_20_ and the nuclei were stained with Hoechst 33342 (1:800; Enzo Life Science, USA) in PBS.PCV2-infected PK-15 cells were analyzed using the confocal fluorescence microscope Leica DMi8. PCV2 positive cells were counted and the result of virus neutralization assay was expressed as percentage of inhibition ratio of PCV2 infection. Assay was performed in 3 independent experiments; 6 areas per well were counted.

### Statistical analysis

Statistical analyses were carried out using GraphPad Prism 8 software. Data were expressed as mean ± SEM. Statistical analyses of differences between groups were performed using one-way ANOVA with Bonferroni post hoc test. Values of *P* < 0.05 were considered statistically significant.

### Ethics approval and consent to participate

All the animal work was conducted according to the principles enunciated in the Guide for the Care and Use of Laboratory Animals issued by the Czech Society for Laboratory Animal Science and the study was approved by the Ethical committee of Ministry of Agriculture of the Czech Republic (permit no. MZE-2334 and 9487/2019-3). All animal experiments were compliant with the ARRIVE guidelines for experimental design and reporting of data.

### Supplementary Information

Supplementary Information.

## Data Availability

All data generated or analyzed during this study are included in this published article.
