# Supplementary material for: UV-C irradiation as an effective tool for sterilization of porcine chimeric VP1-PCV2bCap recombinant vaccine
Source: Sci Rep. 2023 Nov 7;13:19337. doi: 10.1038/s41598-023-46791-9 (PMC10630496; doi:10.1038/s41598-023-46791-9)

**Supplementary information:**

**Figure S1: Raw data to Figure 4A.**


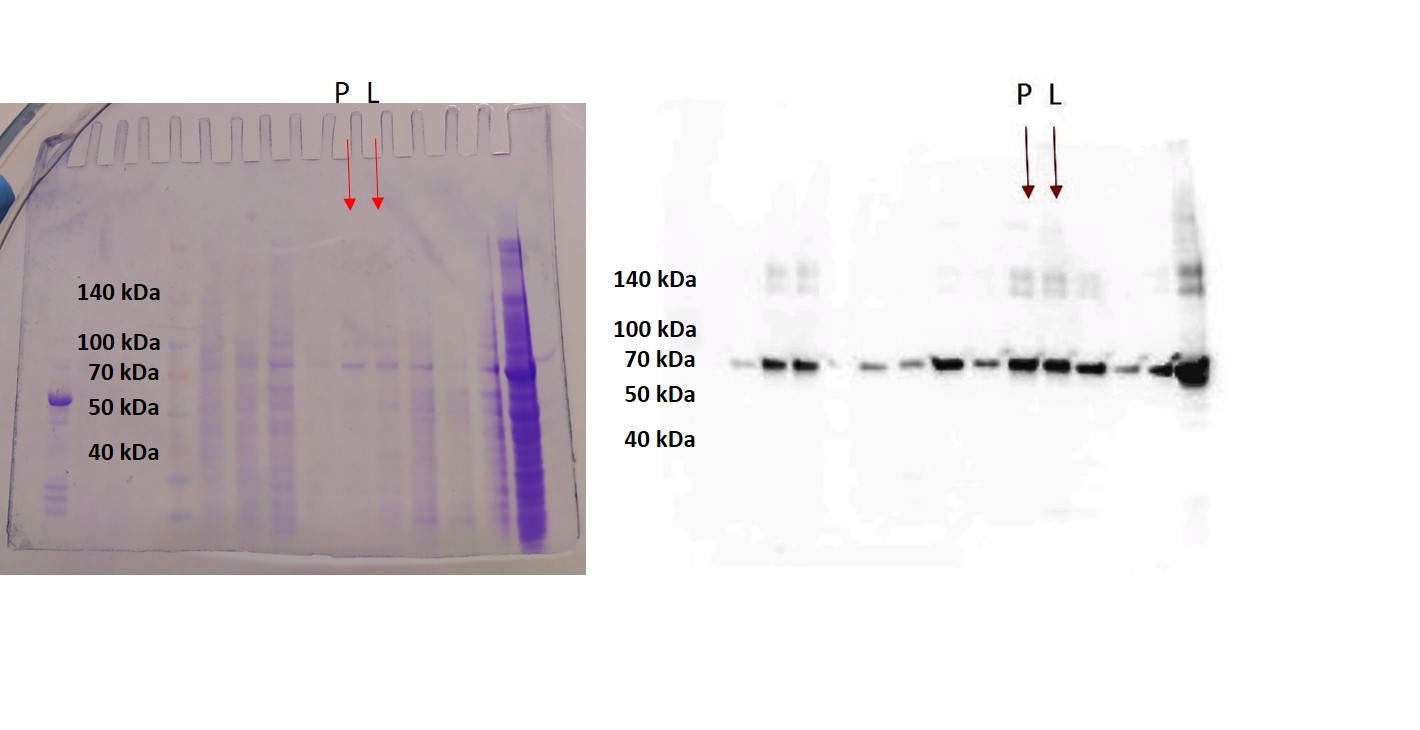


**Figure S2: Raw data to Figure 4B.**


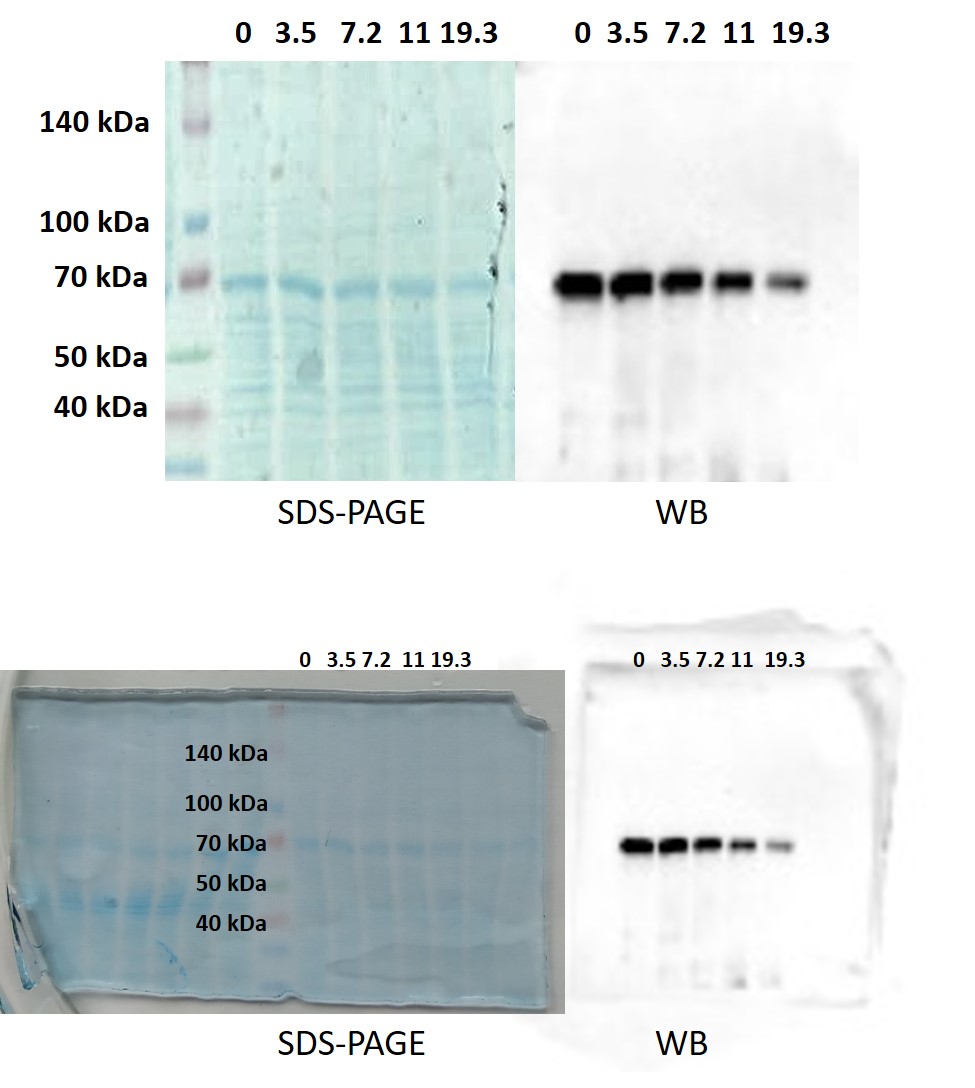


**Figure S3: Significant loss of the purified protein after the filtration.** Purified protein was filtered through syringe filters 0.4 µm or 0.8 µm (Millex AA filter Unit, Merck, Germany) and separated on SDS-PAGE followed by Coomassie Brilliant Blue staining. P – purified protein; F1 – protein filtered through 0.4 µm filter; F2 - protein filtered through 0.8 µm filter


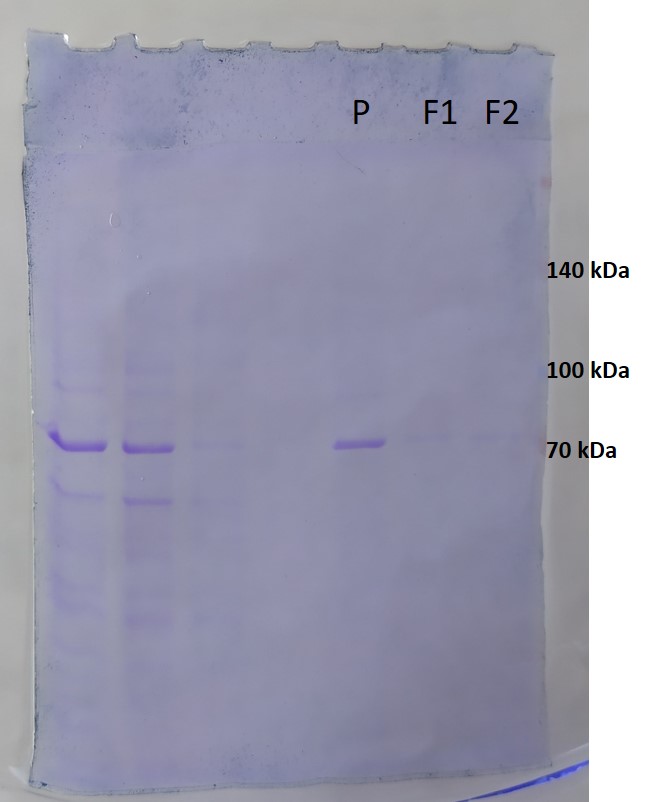

Supplement: Supplementary file 1 — Supplementary Information. [file 41598_2023_46791_MOESM1_ESM.docx]
